# Age-Dependent Enterocyte Invasion and Microcolony Formation by *Salmonella*

**DOI:** 10.1371/journal.ppat.1004385

**Published:** 2014-09-11

**Authors:** Kaiyi Zhang, Aline Dupont, Natalia Torow, Fredrik Gohde, Sara Leschner, Stefan Lienenklaus, Siegfried Weiss, Melanie M. Brinkmann, Mark Kühnel, Michael Hensel, Marcus Fulde, Mathias W. Hornef

**Affiliations:** 1 Institute for Medical Microbiology and Hospital Epidemiology, Hannover Medical School, Hannover, Germany; 2 Department of Molecular Immunology, Helmholtz Centre for Infection Research (HZI), Braunschweig, Germany; 3 Department of Viral Immune Modulation, Helmholtz Centre for Infection Research (HZI), Braunschweig, Germany; 4 Centre for Anatomy, Hannover Medical School, Hannover, Germany; 5 Division of Microbiology, University of Osnabrück, Osnabrück, Germany; University of California, Davis, United States of America

## Abstract

The coordinated action of a variety of virulence factors allows *Salmonella enterica* to invade epithelial cells and penetrate the mucosal barrier. The influence of the age-dependent maturation of the mucosal barrier for microbial pathogenesis has not been investigated. Here, we analyzed *Salmonella* infection of neonate mice after oral administration. In contrast to the situation in adult animals, we observed spontaneous colonization, massive invasion of enteroabsorptive cells, intraepithelial proliferation and the formation of large intraepithelial microcolonies. Mucosal translocation was dependent on enterocyte invasion in neonates in the absence of microfold (M) cells. It further resulted in potent innate immune stimulation in the absence of pronounced neutrophil-dominated pathology. Our results identify factors of age-dependent host susceptibility and provide important insight in the early steps of *Salmonella* infection *in vivo*. We also present a new small animal model amenable to genetic manipulation of the host for the analysis of the *Salmonella* enterocyte interaction *in vivo*.

## Author Summary

Non-typhoidal *Salmonella* are among of the most prevalent causative agents of infectious diarrheal disease worldwide but also very significantly contribute to infant sepsis and meningitis particularly in developing countries. The underlying mechanisms of the elevated susceptibility of the infant host to systemic *Salmonella* infection have not been investigated. Here we analyzed age-dependent differences in the colonization, mucosal translocation and systemic spread in a murine oral infection model. We observed efficient entry of *Salmonella* in intestinal epithelial cells of newborn mice. Enterocyte invasion was followed by massive bacterial proliferation and the formation of large intraepithelial bacterial colonies. Intraepithelial, but not non-invasive, extracellular *Salmonella* induced a potent immune stimulation. Also, enterocyte invasion was required for translocation through the mucosal barrier and spread of *Salmonella* to systemic organs. This requirement was due to the absence of M cells, specialized epithelial cells that forward luminal antigen to the underlying immune cells, in the neonate host. Our results identify age-dependent factors of host susceptibility and illustrate the initial phase of *Salmonella* infection. They further present a new small animal model amenable to genetic manipulation to investigate the interaction of this pathogen with epithelial cells and characterize the early steps in *Salmonella* pathogenesis.

## Introduction

Non-typhoidal *Salmonella* are one of the major causal agents of bacterial gastrointestinal infections in all age groups worldwide. In addition, they also significantly contribute to systemic infection particularly in the pediatric population. Non-typhoidal *Salmonella* together with pneumococci and group B streptococci are among the most frequent causal agents of sepsis and meningitis in neonates and young infants in Africa [1]–[5]. Transmission of this highly endemic bacterium occurs perinatally from infected mothers or after birth from infected family members *via* the fecal-oral route. The underlying mechanisms of the enhanced susceptibility to systemic disease after infection during the postnatal period have not been systematically investigated.

*Salmonella* pathogenicity is conferred by horizontally acquired chromosomal regions so called *Salmonella pathogenicity islands* (SPI) that encode sets of virulence factors required for the various steps in microbial virulence. SPI1 encodes a type III secretion systems (T3SS) and a set of effector molecules that are translocated into the target cell. It mediates pathogen-induced internalization in non-phagocytic cells [6]–[8]. The critical importance of cellular invasion by *Salmonella* and intracellular proliferation in non-phagocytic cells is supported by epidemiological data and has been extensively studied *in vitro* [6], [9]–[10]. Although intracellular *Salmonella* have been observed in epithelial cells and cells of the *lamina propria* also *in vivo*, this host cell interaction and the functional importance have remained less defined [7]–[8], [11]–[15]. Instead, translocation through microfold (M) cells has been demonstrated in adult animals. Such cells overlay Peyer's patches and forward luminal antigens and bacteria to the underlying immune cells [16]–[18]. Similarly, uptake via *lamina propria* resident myeloid cells that generate membrane extrusions extending into the intestinal lumen has been proposed to facilitate *Salmonella* barrier penetration [19]. More recently, rapid translocation *via* the colonic epithelium in the absence of intracellular proliferation was observed [15]. The underlying host and bacterial factors facilitating enterocyte invasion, intraepithelial proliferation and mucosal translocation have, however, remained largely undefined.

Here we comparatively analyzed oral *Salmonella* infection of neonate and adult mice. We demonstrate marked age-dependent differences in intestinal colonization, mucosal translocation and systemic spread. We observed *Salmonella* enterocyte invasion in neonate mice *in vivo* and characterize intraepithelial microcolony formation, epithelial translocation and epithelial innate immune stimulation after oral infection. We characterize developmental peculiarities of the newborn's intestinal epithelium that allow enterocyte invasion and characterize the contribution of *Salmonella* virulence mechanisms. Our results identify age-dependent factors of infection susceptibility and provide a novel *in vivo* model that allows the use of genetically modified hosts to investigate the intimate host microbial interaction at the intestinal epithelium.

## Results

### Mucosal translocation in neonates depends on Salmonella-induced internalization by non-phagocytic cells

**Figure 1:**
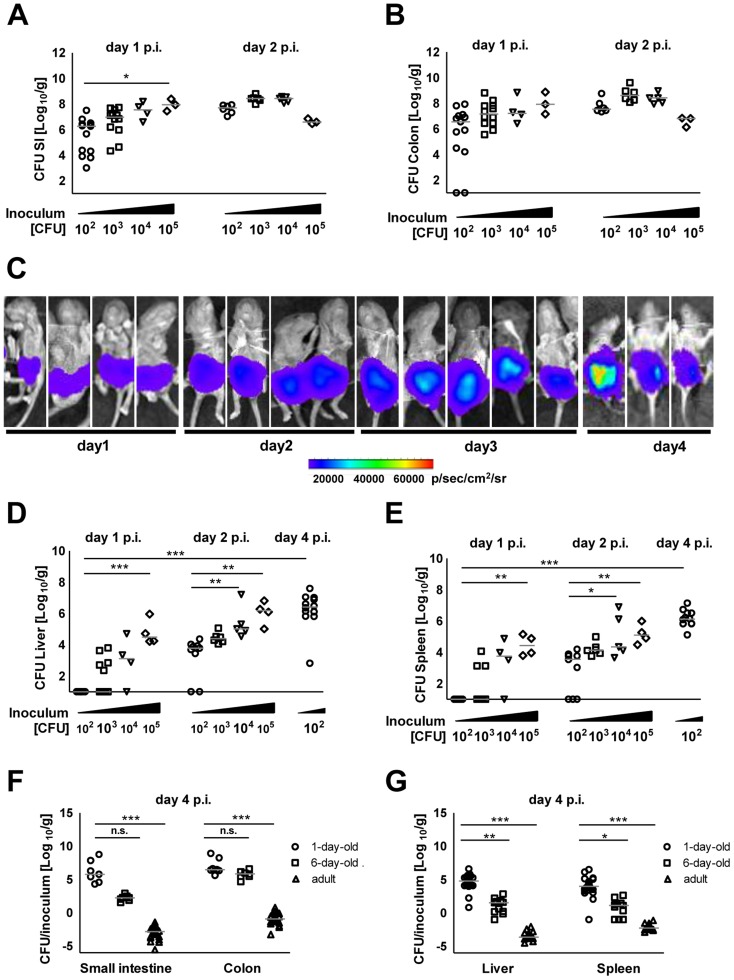
Intestinal colonization and systemic spread of *S.* Typhimurium. Number of viable bacteria in total (**A**) small intestine and (**B**) colon at day 1 and 2 p.i. The results represent the median values from 2–3 independent experiments (n = 3–6 per group). (**C**) Whole body IVIS imaging of 1-day-old mice orally infected with 5×10^3^ CFU of a lux expressing *S.* Typhimurium strain. Strength of the detected light emission in p/sec/cm^2^/sr. 4 mice were used per group in 2 independent experiments. (**D and E**) Number of viable bacteria in total liver (**D**) and spleen (**E**) at day 1, 2, and 4 p.i. with the indicated inoculum. The results represent the median values from 2–3 independent experiments (n = 3–6 per group). (**F and G**) Colonization of the small intestine and colon (**F**) as well as organ counts of liver and spleen (**G**) 4 days after oral infection of 1-day-old (10^2^ CFU), 6-day-old (5×10^2^ CFU) and streptomycin pre-treated adult (6 week-old) mice (5×10^8^ CFU). The values indicate the total organ CFU divided by the inoculum. The results represent the median values from 2–3 independent experiments (n = 3–6 per group).

One-day-old C57BL/6 neonates were orally infected with various inocula (10^2^–10^5^) of mid-log grown *Salmonella enterica* subsp. *enterica* sv. Typhimurium (*S.* Typhimurium). Colony counts and visualization of bioluminescent *S.* Typhimurium in the intestine using optical imaging technology demonstrated rapid colonization of the small intestine and colon (Fig. 1A–C). Even low dose infection (10^2^ CFU) resulted in systemic spread with maximal bacterial counts in spleen and liver at day 4 post infection (p.i.) (Fig. 1D–E). The absence of bacteria in lung tissue excluded accidental primary pulmonary infection (Fig. S1A). Comparative analysis of 1-day-old, 6-day-old and streptomycin pretreated adult animals at day 4 after oral administration of 10^2^, 5×10^2^, and 5×10^8^ CFU *S.* Typhimurium, respectively, demonstrated the efficient colonization of the neonate intestine and the marked spread to liver and spleen tissue in 1-day-old animals (Fig. 1F–G). We therefore focused on 1-day-old animals and applied low dose infection and analyzed animals at 4 days p.i. for all subsequent experiments, if not stated otherwise.

**Figure 2:**
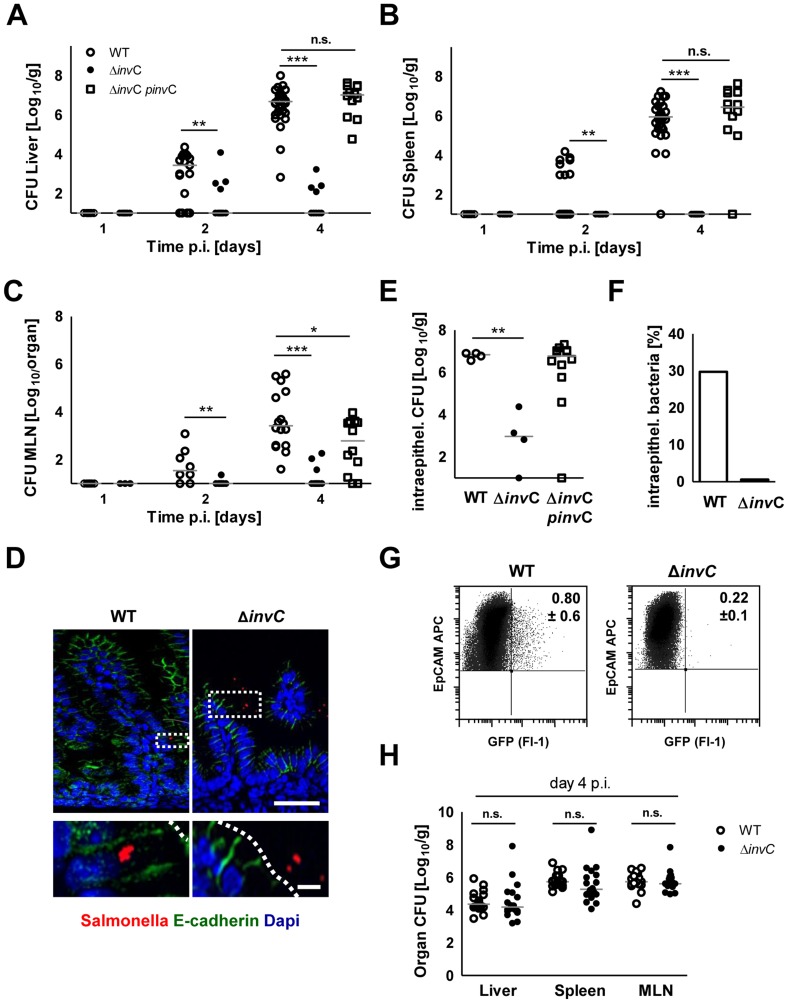
Comparative analysis of WT and invasion-deficient *Salmonella*. (**A–C**) Organ counts in liver (**A**), spleen (**B**) and MLN (**C**) 1, 2 and 4 days after oral infection of 1-day-old mice with 10^2^ CFU WT (open circles), Δ*invC* SPI1 mutant (filled circles) or complemented Δ*inv*C *pinv*C (open squares) *S.* Typhimurium. The results represent the median values from 3–4 independent experiments (n = 9–15 per group). (**D**) Immunostaining of *S.* Typhimurium (red) in small intestinal tissue sections of 1-day-old mice 4 days p.i. with 10^2^ CFU WT or SPI1 mutant (Δ*inv*C) *S.* Typhimurium. Counterstaining with E-cadherin (green) and Dapi (blue). Bar, upper panel = 20 µm; lower panel = 5 µm. (**E**) Viable bacteria cultured from gentamicin treated primary enterocytes isolated at day 4 p.i. with WT or SPI1 mutant (Δ*invC*) *S.* Typhimurium. The results represent the median values from one out of two independent experiments (n = 4–6 per group). (**F**) Median of the number of intraepithelial gentamicin-protected bacteria as shown in (E) divided by the total number of bacteria cultured from untreated isolated IECs (in %). (**G**) Flow cytometric analysis of infected enterocytes (Fl-1) isolated at day 4 after infection of 1-day-old mice with 10^2^ CFU WT or SPI1 mutant (Δ*invC*) (GFP^+^) *S.* Typhimurium. Cells were gated for the epithelial cell marker EpCAM (APC). The number of GFP-positive enterocytes of all EpCAM^+^ cells is indicated (%). One representative data set of three independent experiments is shown. Note that due to the relatively high autofluorescence of enterocytes, cell infected by low numbers of bacteria may remain undetected; the indicated gating might therefore underestimate the number of *Salmonella* infected cells. (**H**) Organ counts in liver, spleen and MLN after oral infection of 6-week-old streptomycin (20 mg) pretreated mice infected with 2×10^8^ CFU WT (open circles, n = 21) or isogenic Δ*invC* SPI1 mutant (filled circles, n = 18) *S.* Typhimurium (n = 6). The results represent the median values from three experiments.

As SPI1 is involved in *Salmonella* internalization in non-phagocytic cells, we next analyzed the requirement of *Salmonella* SPI1 effector translocation for oral infection of neonatal mice. Spread to liver (p<0.001), spleen (p<0.001) and mesenteric lymph nodes (MLN, p<0.001) was practically abolished in the absence of a functional SPI1 system (Δ*invC*) (Fig. 2A–C). In fact, spleen tissue of all mice and liver and MLN tissue of the majority of neonates (8/14 and 18/23, respectively) remained sterile at day 2 and 4 after infection by SPI1 deficient *Salmonella*. Similarly, systemic dissemination of Δ*invC Salmonella* was also highly significantly reduced after high dose (10^5^ CFU) infection (Fig. S2D). In accordance with these results, immunostaining (Fig. 2D), plating of isolated epithelial cells after gentamicin-treatment to remove extracellular *Salmonella* (Fig. 2E–F) and flow cytometry (Fig. 2G) detected enterocytes infected by WT but not SPI1-deficient *Salmonella*. Both, WT and Δ*invC Salmonella* after high and low dose infection spontaneously colonized the intestinal tract (Fig. S2A–C). The reduced total small intestinal organ counts at later points after low dose infection may result from the lack of intraepithelial bacteria (Fig. 2E–F).

### Requirement for enterocyte invasion in the absence of M cells in the neonate intestine

**Figure 3:**
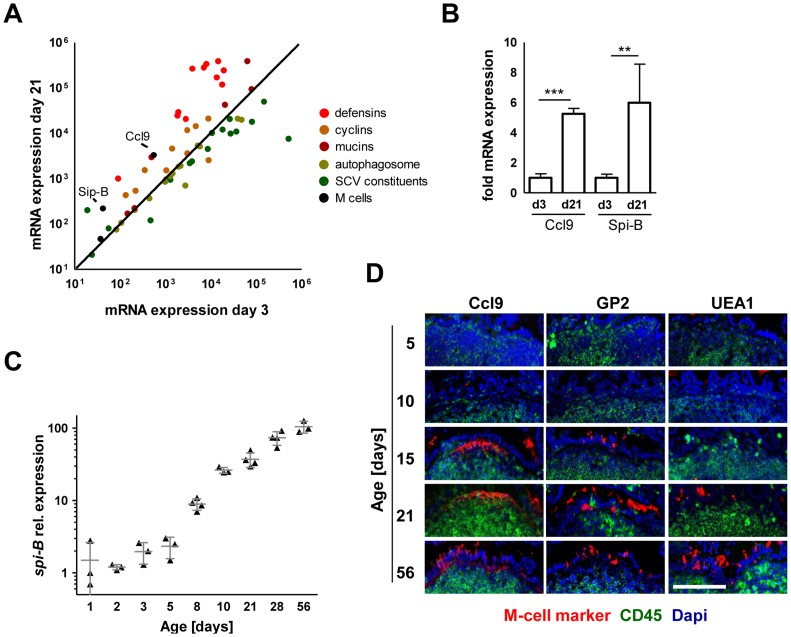
Absence of mature M cells in the neonatal small intestine. (**A**) Comparative gene expression analysis of M cell specific genes (black: Spi-B, Ccl9), antimicrobial peptide or protein genes (red: Defcr3, Defcr4, Defcr6, Defcr20, Defcr22, Defcr23, Defcr-rs1, Defcr-rs7, Defcr-rs12, Reg3g, Saa1), cyclin genes (brown: Ccna2, Ccnb1, Ccnb2, Ccnc, Ccnd1, Ccnd2, Ccnd3, Ccne1, Ccne2, Ccnf), mucin genes (dark red: Muc2, Muc3, Muc4, Muc5b, Muc6, Muc13, Muc20), autophagosome constituents (light green: Atg3, Atg4a, Atg4b, Atg4c, Atg4d, Atg5, Atg7, Atg10, Atg12, Atg16l1, Atg16l2, LC3, p62, NDP52), and SCV constituents (dark green: gp91-phox, p22-phox, iNOS, vATPase, Eea1, Lamp1, Lamp2, Rab5a, Rab5b, Rab5c, Rab7, lysosomal acid phosphatase, mannose-6-phosphate receptor, cathepsin A exopeptidase, cathepsin D endopeptidase, cathepsin L endopeptidase, G6pc, Frap1) obtained from a two-color comparative gene expression array of isolated highly pure primary intestinal epithelial obtained from healthy 3-day-old *versus* 21-days-old C57BL/6 mice. The expression array data are accessible through GEO Series accession numbers GSE35596 and GSE35597. (**B**) Fold mRNA expression analysis of Ccl9 and Spi-B in primary enterocytes from 21-day-old mice as compared to 3-day-old neonate animals. The results represent the mean ± SD (n = 4 per group). (**C**) Quantitative RT-PCR analysis for *Spi-B* mRNA in total enterocytes isolated from healthy C57BL/6 mice at the indicated age (n = 3–4 per group). (**D**) Immunostaining for the M cell markers Ccl9, gp2 and UEA-1 (red) in small intestinal tissue sections obtained from healthy 5-, 10-, 15-, 21-, and 56-day-old adult C57BL/6 mice. Peyer's patches were identified using an anti-CD45 antibody (green). Counterstaining with Dapi (blue). Bar = 100 µm.

In contrast to the critical role of SPI1 in neonate mice, intestinal colonization but also spread to spleen, liver and MLN in adult streptomycin-pretreated animals was largely SPI1-independent (Fig. 2H and Fig. S2E). To identify differences that might account for the requirement of SPI1 in neonate but not adult animals, the gene expression profile of isolated primary neonate and adult epithelial cells was compared. Unexpectedly, genes of differentiated M cells such as Spi-B and Ccl9 were found to be markedly reduced in neonate epithelial cells (Fig. 3A–B). In adult hosts, M cell-mediated bacterial translocation represents the major entry pathway of *Salmonella* [16], [17]. To confirm the age-dependent differentiation of M cells, mRNA expression of the M cell specific transcription factor Spi-B was examined [18]. As expected, Spi-B expression was found to be strongly diminished in neonates and an increase was only observed starting at day 8 after birth (Fig. 3C). Also, immunostaining of intestinal tissue for the established M cell markers glycoprotein 2 (gp2), Ccl9 and Ulex europaeus agglutinin (UEA)-1 confirmed the appearance of M cells only after the neonatal period (Fig. 3D). Finally, wildtype (WT) and *fimD* mutant *Salmonella* disseminated to a similar degree to the spleen and liver tissue after 4 days infection of 1-day-old neonates (Fig. S3A). *fimD* mutant *Salmonella* are unable to express type I pili and cannot attach to the M cell surface protein gp2. This significantly reduces their ability to invade the adult host via M cells [20]. In contrast to a recent report on *Salmonella*-induced Spi-B expression in adult animals, Spi-B expression in neonate mice was reduced rather than enhanced following *Salmonella* infection (Fig. S3B) [21]. Hence, *Salmonella* in neonate mice spontaneously colonize the intestine, invade enterocytes, penetrate the mucosal barrier and spread to systemic organs in a SPI1-dependent but M cell-independent fashion.

### Epithelial invasion leads to intraepithelial proliferation and microcolony formation

**Figure 4:**
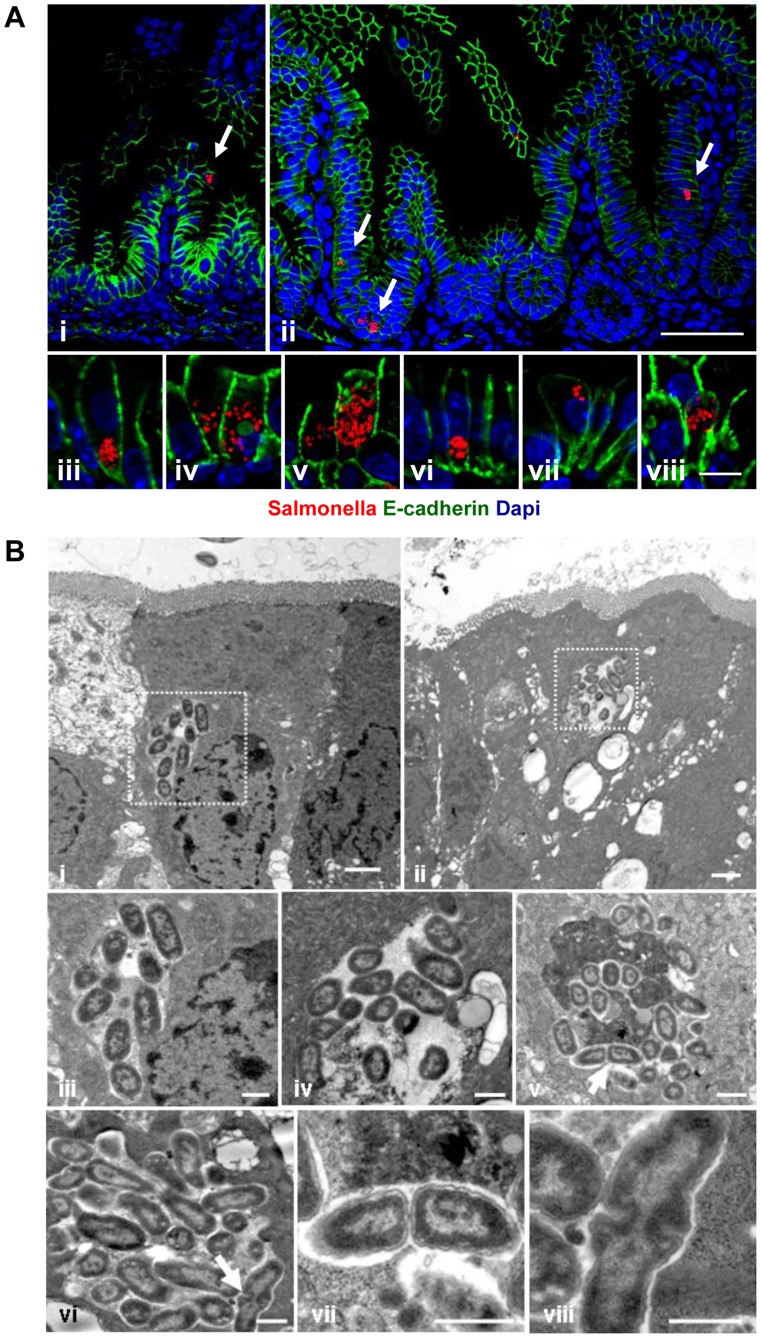
Visualization of intraepithelial *Salmonella*. (**A**) Immunostaining in small intestinal tissue 4 days after 10^2^ CFU *S.* Typhimurium (red) infection of 1-day-old neonate mice. Counterstaining with E-cadherin (green) and Dapi (blue). i and ii, bar = 20 µm; iii–viii, bar = 5 µm. (**B**) Transmission electron microscopy of enterocytes from 1-day-old mice obtained at day 4 p.i. with *S.* Typhimurium. Image iii and iv represent enlarged parts of image i and ii, respectively. Image vii and viii represent enlarged images of the bacteria marked with an arrow in image v and vi, respectively. Bar: i and ii = 2 µm; iii–viii = 1 µm.

**Figure 5:**
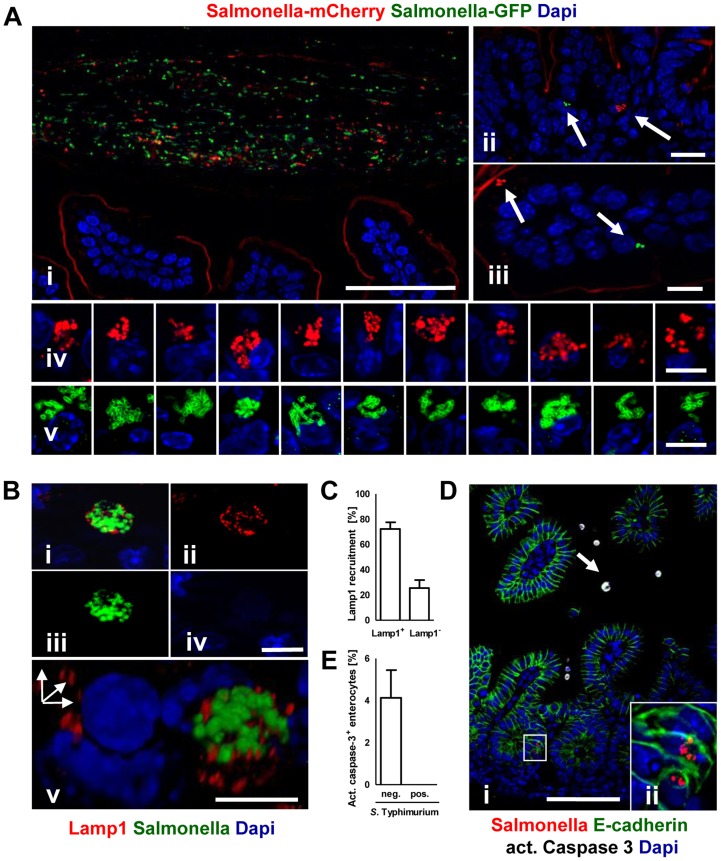
Characterization of *S.* Typhimurium infected enterocytes. (**A**) Immunostaining of C57BL/6 mice simultaneously infected with two genetically labelled *S.* Typhimurium strains orally administrated at a ratio of 1∶1. Staining was performed simultaneously with fluorophore-conjugated anti-GFP (green) and anti-mCherry antibodies (red). Note the presence of equal numbers of both green and red bacteria in the intestinal lumen in (i). Note also that the anti-mCherry antibody cross-reacted to some extend with the epithelial brush border. Counterstaining with Dapi (blue). Bar, i = 50 µm; ii and iii = 10 µm; iv and v = 5 µm. (**B**) Co-immunostaining of the SCV marker Lamp1 (red) and *S.* Typhimurium (green). i represents the merged image, ii–iv single color channel images. v shows a three-dimensional reconstruction to illustrate the punctuate Lamp1 staining surrounding the SCV. Counterstaining with Dapi (blue). Bar, i = 5 µm. (**C**) Quantitative analysis of the percentage of *S.* Typhimurium microcolonies associated with Lamp1 staining. All detected microcolony positive cells in 3 sections obtained from 3 *S.* Typhimurium infected animals were analyzed at day 4 p.i. The results represent the mean ± SD. (**D**) Co-immunostaining for active caspase 3 (white, arrow) and *Salmonella* (red) in small intestinal tissue sections obtained at day 4 p.i. Image ii represents an enlarged part of image i as indicated. Counterstaining with E-cadherin (green) and Dapi (blue). Bar, i = 50 µm. (**E**) Quantitative analysis of the percentage of active caspase 3 positive enterocytes among *S.* Typhimurium-infected and non-infected cells. 20 image areas (Magnification ×20) of intestinal sections from 3 *S.* Typhimurium infected animals were analyzed at day 4 p.i. The results represent the mean ± SD.

*Salmonella* infection of neonatal enterocytes *in vivo* was subsequently studied in more detail. Immunostaining revealed *Salmonella*-positive enterocytes (Fig. 4A). Unexpectedly, *Salmonella* generated multi-bacterial intraepithelial colonies of variable size consistent with the formation of *Salmonella*-containing vacuoles (SCV) previously observed in epithelial cell lines *in vitro* (Fig. 4A–B). Quantification of *Salmonella*-positive cells was achieved by flow cytometric analysis on isolated enterocytes. 0.24±0.12% and 0.80±0.61% of all murine epithelial cell adhesion molecule (EpCAM)^+^ cells at 2 and 4 days p.i., respectively were found to be infected (uninfected controls: 0.07±0.03%, p<0.05). Co-infection using a 1∶1 mixed inoculum of genetically green- and red-labeled bacteria revealed solely single-colored intracellular colonies (Fig. 5A). This suggests that individual microcolonies originated from a single event of bacterial invasion.

Electron microscopy showed that bacteria were enclosed in most instances by a detectable endosomal membrane (Fig. 4B). The intra-endosomal material appeared heterogeneous with hyper- or hypodense areas (Fig. 4Biv–v). Consistently, the well-established SCV transmembrane marker protein lysosomal-associated membrane protein (Lamp) 1 was detected in close proximity to the majority of intraepithelial microcolonies by immunostaining (Fig. 5B–C). In some instances, however, direct contact of individual bacteria with the host cell cytosol could not be excluded. Clear morphological signs of intracellular bacterial proliferation were noted (Fig. 4Bv–viii) in accordance with a rise in the mean fluorescence intensity (MFI) of infected EpCAM^+^ enterocytes from 59.5±35.1 to 98.4±62.1 at 2 and 4 days p.i., respectively. Despite invasion and intracellular proliferation, *Salmonella*-positive cells appeared morphologically intact (Fig. 4B). Whereas a low number of uninfected enterocytes stained positive for active caspase 3, no apoptotic *Salmonella*-positive cells were identified (Fig. 5D–E).

In sharp contrast, only minute numbers of (questionably) intraepithelial *Salmonella* without any indication of bacterial proliferation were detected in tissue sections of adult mice after high dose (1–2×10^8^ CFU) infection despite numerous attempts both with and without streptomycin pretreatment (data not shown). Consistently, flow cytometric analysis did not detect *Salmonella-*infected intestinal epithelial cells (Fig. S2F and). To identify molecular differences that might account for the age-dependent susceptibility of the epithelium to *Salmonella* invasion, the gene expression profile of neonate and adult enterocytes was compared (Fig. 3A). Known SCVs constituents (with the notable exception of iNOS) and autophagosomal markers were equally expressed in neonate and mature adult epithelial cells. However, the neonate intestine exhibited a markedly reduced mucus layer thickness and mucin glycoprotein expression (Fig. S3C–D). Also, reduced cyclin expression was noted in accordance with minimal epithelial cell turn-over and reduced crypt-villus migration in neonates [22], [23]. Finally, a severely reduced spectrum of antimicrobial peptides was observed consistent with previous reports [24]. All of these factors might facilitate epithelial invasion, prolong the lifetime of infected epithelial cells and thereby ultimately allow intraepithelial proliferation and microcolony formation.

### Invasion-dependent innate immune stimulation of epithelial cells

**Figure 6:**
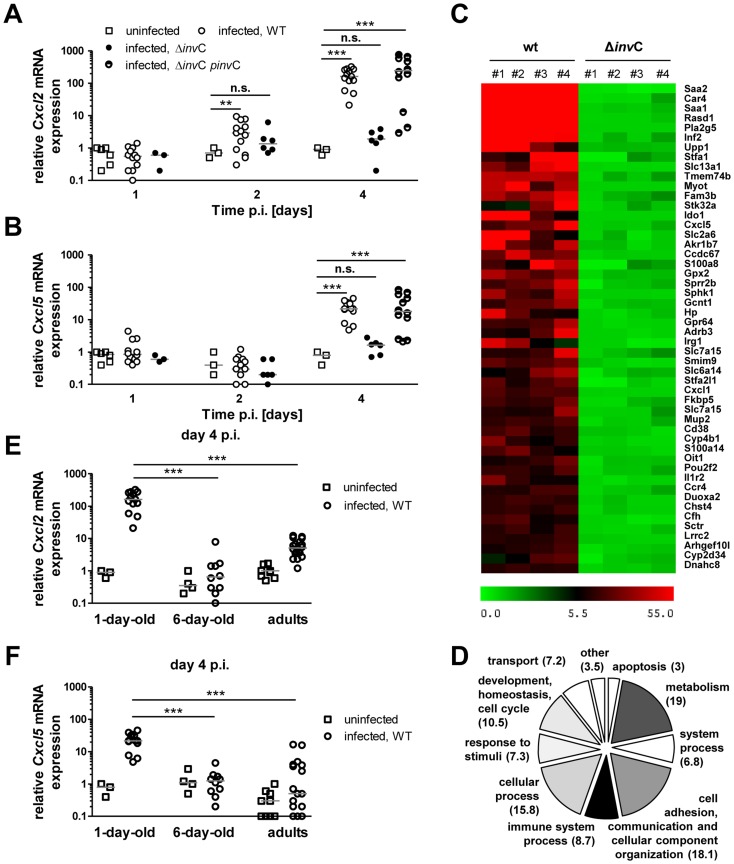
Invasion-associated epithelial innate immune stimulation. (**A and B**) Quantitative RT PCR for (**A**) *Cxcl2* and (**B**) *Cxcl5* mRNA in enterocytes isolated at 1, 2 and 4 days p.i. with 10^2^ CFU WT (open circles, n = 13), Δ*invC* SPI1 mutant (filled circles, n = 6) or complemented Δ*invC* p*invC* (semi-filled circles, n = 12) *S.* Typhimurium. The results represent the median values from 2–5 independent experiments (n = 6–13 per group). (**C**) Heat map of the relative gene expression in enterocytes of each 4 individual WT or SPI1 mutant (Δ*invC*) *S.* Typhimurium infected animals (#1–#4) as compared to 4 non-infected control animals (fold increase). The 50 most highly expressed genes following WT *S.* Typhimurium infection are shown. (**D**) COG analysis of >2.5-fold induced genes in primary isolated intestinal epithelial cells following low dose (10^2^ CFU) wildtype *S.* Typhimurium infection. (**E and F**) Quantitative RT PCR for (**A**) *Cxcl2* and (**B**) *Cxcl5* mRNA in enterocytes isolated at 4 days p.i. after infection of 1-day-old neonate (1–2×10^2^ CFU), 6-day-old neonate (5×10^2^ CFU), 6 week-old adult (2×10^8^ CFU) mice with WT *S.* Typhimurium (open circles) or uninfected age-matched control animals (open squares). The results represent the median values from three independent experiments (n = 3–6 per group).

We next investigated the neonate's host response to *Salmonella* infection. Highly enriched primary enterocytes (Fig. S4) were subjected to quantitative RT-PCR. A significant time-dependent increase in *Cxcl2* and *Cxcl5* mRNA expression was measured after infection by WT or the complemented Δ*invC* p*invC* but not SPI1-deficient non-invasive *Salmonella* (Fig. 6A–B). Global gene array analysis confirmed the absence of any detectable increase in epithelial gene expression upon administration of *invC* deficient, non-invasive *Salmonella* (Fig. 6C). It further identified a large number of additional genes involved in metabolism, cellular responses and intercellular communication induced after *Salmonella* WT infection (Fig. 6D). In accordance with the requirement of enterocyte invasion for innate immune stimulation (Fig. 6C) and the observed age-dependent susceptibility to enterocyte invasion (Fig. 2D–G and S2F), *Cxcl2* and *Cxcl5* mRNA expression in enterocytes isolated at day 4 p.i. from infected 6-day-old or adult mice was severely reduced (Fig. 6E–F).

**Figure 7:**
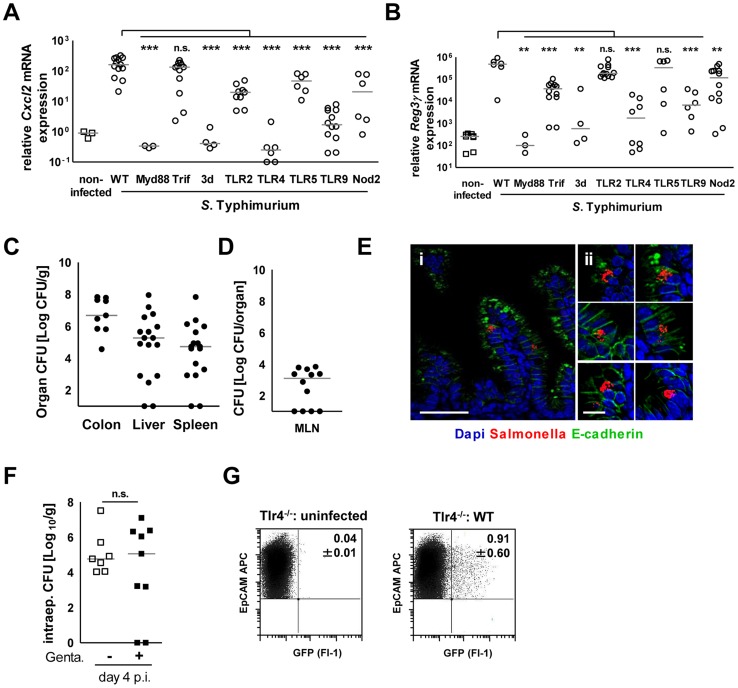
Epithelial innate immune stimulation during neonatal *Salmonella* infection. (**A and B**) Quantitative RT PCR for (**A**) *Cxcl2* and (**B**) *Reg3γ* mRNA in enterocytes isolated from the indicated gene-deficient mice at 4 days p.i. with 10^2^ CFU WT *S.* Typhimurium. The results represent the median values from 2–3 independent experiments (n = 3–14 per group: wt, n = 14; Tlr4^−/−^, n = 9; Tlr5^−/−^, n = 6; Myd88^−/−^, n = 3; Trif^−/−^, n = 12; TLR2^−/−^, n = 12; TLR9^−/−^, n = 12; 3d (Unc93B1 H412R), n = 3; NOD2^−/−^, n = 12). (**C and D**) Organ counts in (C) colon, spleen, liver and (D) mesenteric lymph nodes (MLN) of 1-day-old Tlr4^−/−^ neonate mice after oral infection with 10^2^ CFU WT *S.* Typhimurium. The results represent the median values from 3 independent experiments (n = 3–6 per group). (**E**) Immunostaining of small intestinal tissue 4 days after infection of 1-day-old Tlr4^−/−^ neonate mice with 10^2^ CFU *S.* Typhimurium (red). Counterstaining with E-cadherin (green) and Dapi (blue). i, bar = 20 µm; ii, bar = 10 µm. (**F**) Viable bacteria cultured from primary enterocytes isolated from small intestinal tissue at day 4 p.i. with WT *S.* Typhimurium and left untreated or incubated in gentamicin. The results represent the median values from one out of two independent experiments (n = 7–9 per group). (**G**) Flow cytometric analysis of enterocytes isolated at day 4 p.i. from 1-day-old Tlr4^−/−^ mice infected with 10^2^ CFU WT (GFP^+^) *S.* Typhimurium or from age-matched control animals. Cells were gated for the epithelial cell marker EpCAM (APC). The number of GFP-positive enterocytes of all EpCAM^+^ cells is indicated (%). One representative data set of three independent experiments is shown.

**Figure 8:**
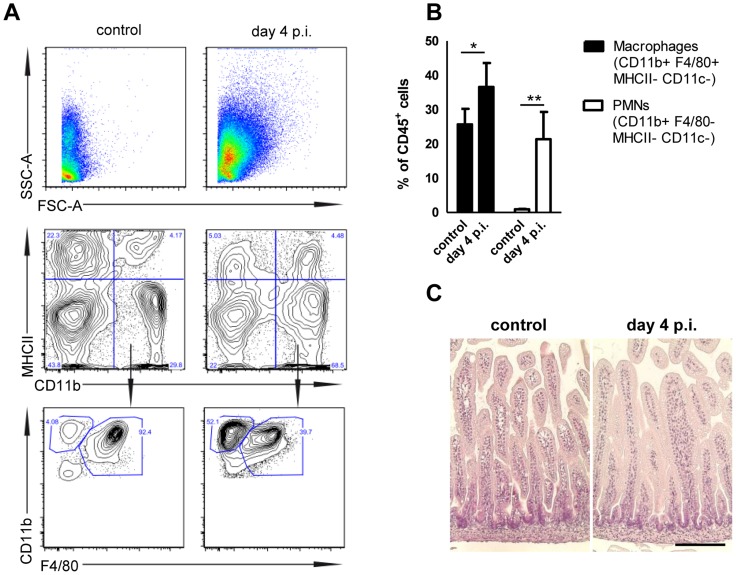
Mucosal tissue response following *S.* Typhimurium infection in neonate mice. (**A**) Flow cytometric analysis of *lamina propria* myeloid immune cells prepared from 1-day-old neonates infected for 4 days with 10^2^ CFU *S.* Typhimurium or non-infected age-matched control animals. Representative images are shown. (**B**) Quantitative analysis of CD11b^+^ F4/80^+^ MHCII^−^ CD11c^−^ tissue invading macrophages and CD11b^+^ F4/80^−^ MHCII^−^ CD11c^−^ polymorphonuclear cells relative to total CD45^+^ cells in the *lamina propria* of 1-day-old neonates infected for 4 days with 10^2^ CFU *S.* Typhimurium or non-infected age-matched control animals. The results represent the mean ± SD from 2 independent experiments (n = 4). (**C**) Hematoxilin and eosin staining of intestinal tissue sections of neonatal mice (small intestine) left untreated or 4 day p.i. with *S.* Typhimurium (10^2^ CFU). Bar = 50 µm.

We next examined the innate immune receptors and signaling adaptors involved in *Salmonella*-induced enterocyte stimulation. Expression of Cxcl2 and Reg3γ mRNA by epithelial cells was severely reduced in the absence of toll-like receptor (Tlr)4, MyD88 as well as Unc93B1 and Tlr9. Although some variability in the expression levels was noted, significantly decreased mRNA expression was also noted in epithelial cells devoid of Tlr2, Tlr5, or Nod2 (Fig. 7A–B). Of note, intestinal colonization (Fig. 7C), enterocyte invasion (Fig. 7E–G) and dissemination to systemic organs (Fig. 7C–D) was observed also in the absence of the most potently stimulated innate immune receptor, Tlr4. Although the production of proinflammatory mediators significantly enhanced the recruitment of polymorphonuclear cells and blood macrophages to the site of infection, no gross loss of epithelial barrier integrity or histopathological signs of tissue destruction were observed at day 4 p.i. in contrast to the situation in the adult host (Fig. 8) [25]. Thus, *Salmonella* in the neonate efficiently invades small intestinal enterocytes, activates innate immune responses mainly *via* Tlr stimulation and induces the formation of intraepithelial microcolonies, a hallmark of the *Salmonella-*enterocyte interaction.

## Discussion

Previous work had demonstrated enterocyte invasion in oral and intestinal loop infection models and *ex vivo* tissue explants of calfs, rabbits, swine and guinea pigs [26]–[32]. However, these host animals are not amenable to genetic manipulation and studies are restricted to the early course of infection. Enterocyte invasion, rapid egress at the basolateral side of the epithelium and the presence of Salmonella in *lamina propria* cells has also been described in the mouse large intestine [7], [11]–[15]. The present study now provides a new small animal model that allows the use of genetically modified hosts to analyse intracellular proliferation in small intestinal epithelial cells and the formation of microcolonies by *Salmonella in vivo*. It might thereby facilitate a better understanding of a hallmark of *Salmonella* pathogenesis *in vitro*, the internalization by non-phagocytic epithelial cells and the formation of intraepithelial microcolonies, so called *Salmonella* containing vacuoles (SCVs).

Invasion of non-phagocytic epithelial cells is facilitated by virulence factors encoded by the pathogenicity island SPI1 [6]. The critical role of SPI1 for enteric disease is illustrated by its presence in all subspecies of *Salmonella enterica* [33] and its high prevalence in clinical *Salmonella* isolates [9]. In accordance, SPI1 effector proteins are required to cause diarrhea and mucosal inflammation in several *in vivo* models although also SPI2 effector molecules contribute to intraepithelial survival and proliferation [25], [34]–[36]. The analysis of the interaction of *S.* Typhimurium with polarized epithelial cells within their anatomical environment represents a prerequisite to understand the functional role of individual bacterial virulence factors.

Whereas previous reports using streptomycin pretreated adult animals failed to demonstrate infection of the small intestinal epithelium, we observe efficient *Salmonella* invasion of enterocytes in neonate mice. Known host constituents of mature SCVs and autophagosome factors are similarly expressed by neonatal and adult epithelial cells [37]–[39]. However, a number of fundamental differences exist between the neonate and adult gut epithelium in mice. For example, we demonstrate that the synthesis of various mucin glycoproteins and thus the thickness of the mucus layer are significantly enhanced in adult animals. The mucus layer was shown to significantly impair mucosal translocation of *Salmonella* in adult mice [40]. Also, we and others have shown that the antimicrobial peptide repertoire is severely reduced in the neonate intestine in the absence of mature Paneth cells. In adult mice, Paneth cell-derived antimicrobial peptides cells significantly influence the course of enteropathogen infection [41], [42] and cooperate with the mucus layer to generate an antibacterial and antiinflammatory shield [43]. Finally, the well-established constant renewal of the epithelium associated with enterocyte migration and exfoliation at the villus tip is not yet established in newborn mice [22], [23]. The reduced epithelial cell turn-over is the consequence of the lack of crypts and the reduced pool of rapidly proliferating cells during this early developmental stage. Thus, enterocytes during the neonatal period remain longer at the same anatomical position. This might allow *Salmonella* to proliferate intracellularly and form microcolonies. Shedding of *Salmonella*-infected enterocytes at the villus tip was previously observed in bovine, pig and rabbit intestinal loop models and might represent an important mechanism to remove infected enterocytes [28], [30], [44]. Finally, the prolonged presence of the more diverse enteric microbiota in the adult host may contribute to the enhanced resistance to *Salmonella* enterocyte invasion and this issue requires future investigations.

In the neonate host both *Salmonella* enterocyte invasion and mucosal translocation were dependent on SPI1. This suggests that penetration of the neonate's intestinal barrier occurs secondary to the observed enterocyte invasion. This is in contrast to the situation in adult mice but also other species. Here, M cells were shown to represent the major port of entry [16]–[18], [29]. M cells as part of the follicle-associated epithelium overlaying Peyer's patches facilitate uptake of particulate antigen [17], [18], [45]. Thus, translocation takes place independent of bacteria-induced internalization [16]. In contrast, the absence of M cells in the neonate mouse intestine shifts the major entry pathway to enterocyte invasion. It thereby pronounces the requirement for SPI1 for mucosal translocation. This may also explain the recent finding that *Yersinia enterocolitica* translocation is highly reduced in neonate mice [46]. Mucosal translocation of enteropathogenic *Yersinia* is also mostly mediated through M cell transport [47]. However, we cannot formally exclude that additional host factors also contribute to the enhanced requirement for a functional SPI1 system in the neonate host.

Strong innate immune receptor-mediated stimulation of the epithelium was noted upon infection in accordance with previous analyses of infected total mucosal tissue [48], [49]. The present study provides a global gene expression analysis of isolated primary enterocytes after *Salmonella* infection *in vivo*. The restriction of innate immune stimulation to invasion-competent *Salmonella* is consistent with reports on the requirement of SPI1 for mucosal inflammation and clinical disease [25], [34]–[35], [49]. Epithelial stimulation was mediated by recognition of *Salmonella* lipopolysaccharide (LPS) through Tlr4 and signaling via MyD88 consistent with previous reports on the involvement of Tlr4 in the adult host defense against *Salmonella* [50], [51]. Similarly, deficiency in Tlr9 and the processing molecule Unc93B1 significantly reduced the epithelial cell response to *Salmonella* infection. The strong effect observed in 3d UNC93B1 mutant mice indicates the possible involvement of additional UNC93B1 dependent innate immune receptors such as Tlr3, 7 or 13 [52]. Our results indicate the synergistic action of Tlr4 and Tlr9 in the epithelial response to *Salmonella* in accordance with previous reports [53]. A minor but significant role was also found for Tlr2, Tlr5 and Nod2 previously implicated in the antimicrobial response of the adult host to *Salmonella* [54]. The synergistic stimulation of Tlr2, 4 and 9 was shown to be required for endosomal acidification and SPI2 effector protein translocation in bone marrow-derived macrophages [53]. A similar scenario may apply to intestinal epithelial cells. In accordance, lack of Tlr4 alone did not significantly influence enterocyte invasion or intraepithelial proliferation *in vivo*. Further studies are required to investigate the role of cooperative immune signaling by different receptors for the process of enterocyte infection and microcolony formation.

Colonization of the neonate intestine occurred largely independent of the invasion-dependent innate immune stimulation consistent with a low degree of colonization resistance in the neonate host. Reduced organ counts in the total small intestine after low dose infection of SPI1 mutant *Salmonella* might result from the lack of proliferating intraepithelial *Salmonella*. Alternatively, invasion-induced immune stimulation might promote metabolic changes that favor pathogen colonization of the neonate intestine as recently described in adult animals [55]. In accordance, a number of metabolic genes were significantly influenced during bacterial challenge supporting the idea of a heavily altered intestinal host metabolism during neonatal infection [56]. In addition, several innate immune response genes such as the chemokines, serum amyloid proteins, iNOS, calprotectin, mucins and antimicrobial lectins were strongly upregulated in infected epithelial cells in accordance with previous reports [57]. As a consequence, large numbers of polymorphonuclear cells (PMNs) but also macrophages were recruited to the site of infection. The cellular immune response at this time point, however, did not lead to significant tissue alteration. The anti-apoptotic effect of NF-κB stimulation might have prevented the induction of epithelial apoptosis previously reported in both *Salmonella*-infected and non-infected enterocytes [44].

In conclusion, we analyzed age-dependent differences in the interaction of *Salmonella* with the host epithelium. We identify host and bacterial factors responsible for the infection process in the neonate host and characterize the epithelial innate immune response. Our results demonstrate enterocyte invasion and intracellular proliferation of *Salmonella* in differentiated and polarized epithelial cells and established a new animal model amenable to genetic manipulation to study the enterocyte-pathogen interaction *in vivo*. However, neonate mice exhibit an immature mucosal immune system, which might contribute to the observed phenotype and requires further characterization. Also, limitations such as the small animal size and lack of a suitable anesthesia required for intravital microscopy as well as the possible exchange of bacteria between newborn mice and the dam exist. Nevertheless, we believe that the described model opens new avenues of research to unravel the functional role of individual effector proteins in the *Salmonella*–enterocyte interaction and to better understand the cellular and immunological events of the *Salmonella* pathogenesis.

## Materials and Methods

### Bacterial strains

The isogenic strain MvP818 harboring a deletion of *inv*C has been described before [58]. For complementation of the *inv*C mutation, plasmids p3545 was generated as follows: The promoter of *inv*F was amplified from *S.* Typhimurium genomic DNA using PinvF-For-EcoRI (CCGGAATTCTCCATCCAG ATGACAATATC) and PinvF-Rev-SmaI (ATATCTAGATCCATCCAGATG ACAATATCTG). The resulting product was digested by EcoRI and SmaI and subcloned in pWSK29 to obtain p3537. *inv*C was amplified using *inv*C-For–SmaI (atacccgggtttagtcg gtcgctaatgag) and *inv*C-Rev-XbaI (GTATCTAGATTAAT TCTGGTCAGCGA ATGC), the resulting fragment was subcloned as SmaI/XbaI fragment in p3537 to generate p3545. Correct clones were confirmed by DNA sequencing and functional complementation of the invasion defect of MvP818 was verified in non-phagocytic intestinal epithelial m-IC_cl2_ cells (Fig. S5A–C). *S.* Typhimurium ATCC 14028 and SPI-1 mutant *S.* Typhimurium MvP813 Δ*inv*C (Kana^R^) carrying a green fluorescent protein (GFP) expression plasmid (*p*GFP, Amp^R^, kindly provided by Brendan Cormack, Stanford, USA) and *S.* Typhimurium ATCC 14028 carrying a mCherry expression plasmid (*p*FPV-mCherry, Amp^R^, kindly provided by Leigh Knodler, NIH, Hamilton, USA) were used for *in vivo* infection experiments. Maintenance of the plasmid *p*GFP in *S.* Typhimurium under *in vivo* conditions was confirmed by comparative plating of spleen and liver tissue simultaneously on selective (100 µg/mL ampicillin) and non-selective LB agar plates (Fig. S6C–D). For oral infection with two different *Salmonella* strains, a 1∶1 mixture of 10^2^ *p*GFP and 10^2^ *p*FPV-mCherry carrying bacteria were administered orally in 2 µl PBS. Life imaging (IVIS) was performed employing an isogenic *S.* Typhimurium strain with a chromosomal insertion of the lux-operon from *Photorhabdus luminescens* under the control of the constitutive ß-lactamase promoter.

### Infection experiments

Adult C57BL/6 wildtype and B6.B10ScN-Tlr4^lps-del^/JthJ Tlr4 (stock no. 007227), B6.129S1-Tlr5^tm1Flv^/J Tlr5 (stock no. 008377), B6.129-Tlr2^tm1Kir^/J (stock no. 004650), and B6.129S1-Nod2^tm1Flv^/J Nod2 (stock 005763) deficient, as well as C57BL/6J-Ticam1^Lps2^/J TRIF mutant (005037) mice were obtained from the Jackson Laboratory (Bar Harbour, USA). 3d [59] and B6.129P2-Tlr9^(tm1Aki)^ Tlr9 deficient mice [60] were obtained from M. Brinkmann, Helmholtz Center for Infection Biology, Braunschweig, Germany. Mice were housed under specific pathogen-free conditions and handled in accordance with regulations defined by FELASA and the national animal welfare body GV-SOLAS (http://www.gv-solas.de).

*Salmonella enterica* subsp. *enterica* serovar Typhimurium ATCC14028 (NCTC12023) WT and isogenic mutant strains were cultured in Luria Bertani (LB) broth overnight at 37°C, diluted 1∶10 and incubated at 37°C until reaching the logarithmic phase (OD_600_ approximately 0.5). Bacteria were washed and adjusted to OD_600_ 0.55–0.60 containing approximately 1.5–2.0×10^8^ CFU/mL and diluted to obtain the appropriate infection dose. All experiments with neonatal mice were performed using 1-day-old C57BL/6 animals. Neonates were infected orally with the indicated number of *S.* Typhimurium in a volume of 1 µl PBS. The administered inoculum was confirmed by serial dilution and plating. *In vivo* imaging was performed using the IVIS200 imaging system (PerkinElmer). For image analysis and visualization the LivingImage 4.3.1 software was used. Oral infection of 6 week-old adult female mice was performed as previously described [25]. Bacterial counts were obtained after homogenization of spleen, liver and mesenteric lymph nodes by serial dilution and plating.

### Ethics statement

All animal experiments were performed in compliance with the German animal protection law (TierSchG) and approved by the local animal welfare committee (approval 12/0697, 12/0693, 13/1097 and 14/1385 of the Niedersachsische Landesamt für Verbraucherschutz und Lebensmittelsicherheit Oldenburg, Germany).

### Primary cell isolation and flow cytometry

Primary intestinal epithelial cells were isolated from small intestinal tissue after incubation in 30 mM EDTA PBS at 37°C for 10 min as described previously [61]. Cells were passed through a 100 µm nylon cell strainer (BD Falcon), washed with 10% FCS/PBS, and harvested by centrifugation. The purity was verified by flow cytometry (Fig. S4). In order to determine the number of cell-associated *versus* intracellular bacteria, one fraction of epithelial cells was plated directly (total number of epithelium-associated bacteria); another fraction was incubated in 100 µg/mL gentamicin for 1 h prior to plating (number of gentamicin-protected, intracellular bacteria). For flow cytometry analysis of intracellular *Salmonella*, cells were fixed with 4% PFA for 15 min. on ice. The cells were washed, stained with APC-conjugated anti-EpCAM (anti-CD326 clone 48.8, diluted 1∶500, from eBioscience) and finally resuspended in 5% FCS/PBS. Prior to analysis, cells were filtered through a 35 µm pore size BD Falcon Polystyrene tube (BD, cat. no. 352235). 300,000 events were acquired and GFP expression was detected using a FACS Calibur apparatus (BD). For purity confirmation of isolated primary enterocytes, cells were fixed and incubated on ice with APC-conjugated anti-EpCAM (clone 48.8) and PE-conjugated anti-CD45 antibodies (clone 30-F11, dilution: 1∶200) purchased from eBioscience, for 30 minutes. Cells were washed and filtered. 25,000 events were acquired. Postacquisition analysis of FACS data was performed using the Summit 5.0 software. For flow cytometric analysis of tissue invading myeloid cells, small intestines from neonatal mice were removed and opened longitudinally. Tissue was digested in Liberase (Roche)/DNAse I (Roche)/10%FCS/RPMI at 37°C and separated on a 40%/70% Percoll gradient. Cell were stained with PerCP Cy5.5 conjugated anti-CD45 (Clone 104), Brilliant Violet-conjugated anti-I-A/I-E (Clone M5/114.15.2), APC/Cy7-conjugated anti-CD11c (Clone N418, all purchased from Biolegend), PE-conjugated anti-CD11b (Invitrogen) and APC-conjugated anti-F4/80 (Clone BM8, from eBioscience). The samples were acquired on a LSRII (BD) and analyzed with FlowJo (Treestar).

### Immunostaining

Immunostaining using a chicken anti-GFP (dilution 1∶500), a mouse anti-mCherry (diluted 1∶500, both from Abcam), a mouse anti *Salmonella* O4-antigen (diluted 1∶500, from Abcam), a rat anti-Lamp1 (diluted 1∶500, from the Developmental Studies Hybridoma Bank (DSHB), University of Iowa, US), a rabbit anti-active Caspase 3 (diluted 1∶200, from Cell Signaling), a rabbit anti-Muc2 (diluted 1∶100, generous gift from Gunnar Hansson, Göteborg, Sweden) and a mouse anti-E-cadherin (diluted 1∶100, from BD Transduction Laboratories), was performed on 3 µm paraformaldehyde-fixed paraffin-embedded tissue sections in combination with the appropriate fluorophore conjugated secondary antibody (Jackson ImmunoResearch). Sections were deparaffinized in xylene and rehydrated in ethanol followed by antigen retrieval in 10 mM sodium citrate and blocking with PBS 10% serum 5% BSA. Staining of constitutively GFP expressing bacteria was performed to enhance the sensitivity of detection. In addition, this approach ensured bacterial detection also under low oxygen conditions, which might be present within the intestinal lumen and have been reported to alter GFP emission. The accuracy of the anti-GFP staining to detect plasmid-bearing *S.* Typhimurium was verified by staining of two consecutive sections cut from the same tissue block with anti-GFP and a biotinylated 1∶100 diluted rat monoclonal anti-O4/O5 antibody generously provided by M. Kim (Kim Laboratories, Champaign, IL). Both staining methods revealed a very similar staining pattern suggesting that the anti-GFP staining method used was both sensitive and specific (Fig. S6A–B). Staining with the rhodamine-conjugated lectin UEA-1 (Vector), a rat anti-GP2 (diluted 1∶100, from MBL) or a goat anti-Ccl9 (diluted 1∶100, from R&D) was performed on 8 µm sections obtained from freshly frozen OCT (Tissue-Tek) embedded tissue. Sections were dried and fixed using methanol at −20°C for 10 min. followed by rehydration in PBS for 15 min. Blocking in 0.2% BSA, 0.2% saponin, 10% serum in PBS was performed prior to immunostaining. Peyer's patches were visualized using a FITC-conjugated anti-CD45 antibody (clone 30-F11, BioLegend). Hematoxylin and eosin staining of paraformaldehyde-fixed tissue sections was performed according to Mayer's protocol using reagents from Roth. Slides were mounted in Vectashield (Vector) supplemented with DAPI and pictures were taken with an Apo-Tome microscope connected to a digital camera (Zeiss).

### Electron microscopy

For electron microscopical analysis of infected intestinal tissue, samples were fixed in 150 mM HEPES, pH 7.35, containing 4% formaldehyde and 0.1% glutaraldehyde at room temperature for 1 hour and then stored over night in fixative at 4°C. Samples were dehydrated in acetone and embedded in EPON. 60 nm sections were mounted onto formvar-coated copper grids, stained with 4% uranyl acetate and lead citrate as previously described by Reynolds and visualized in a Morgagni TEM (FEI), operated at 80 kV [61].

### Gene expression analysis

Total RNA was extracted from isolated enterocytes using TRIzol (Ambion) and the RNA concentration was determined on a NanoDrop 1000 spectrophotometer (Thermo Scientific). First-strand complementary DNA (cDNA) for quantitative RT-PCR was synthesized from 5 µg of RNA with Oligo-dT primers and RevertAid reverse transcriptase (Fermentas). Taqman technology based RT PCR was performed using absolute QPCR ROX mix (Thermo Scientific), sample cDNA and the Taqman probes *hprt* (Mm00446368_m1), *Spi-B* (Mm03048233-m1) *Cxcl2* (Mm00436450_m1), *Cxcl5* (Mm00436451_g1) and *Reg3γ* (Mm01181783_g1) from Life Technologies. SYBR green–based real-time PCRs were performed as described in the Extended Experimental Procedures. Microarray analysis was performed using Whole Mouse Genome Oligo Microarray v2 (4×44k) (Agilent Technologies) following the SC_AgilentV5.7 protocol provided by the manufacturer. Heat map analysis was performed using the MeV 4_5_1 software. Clusters of orthologous groups (COG) analysis was performed using the online bioinformatic tool PANTHER (http://www.pantherdb.org/). The expression array data are accessible through GEO Series accession numbers GSE51160. Reviewer can access the raw data files using the following link: http://www.ncbi.nlm.nih.gov/geo/query/acc.cgi?token=ehufucgejpwtjwf&acc=GSE51160.

### *In vitro* cell culture

Intestinal epithelial m-IC_cl2_ cells were cultured for 6 days with medium change every other day to obtain a confluent cell monolayer as previously described [62]. m-IC_cl2_ cells were incubated with wildtype *S.* Typhimurium, the SPI1 mutant strain (Δ*invC*) or the complemented SPI1 mutant strain (Δ*invC* p*invC*) at a multiplicity of infection of 1∶10 for 1 h at 37°C, washed and gentamicin (Sigma Aldrich) was added at a concentration of 100 µg/mL for 1 h at 37°C. Cells were washed 3 times in PBS and lysed directly or maintained in culture in the presence of 20 µg/mL gentamicin. At the indicated incubation period cells were washed with PBS and lysed in 500 µl 0.1% Triton X-100 for 2 min. at room temperature. Viable counts were determined by serial dilution and plating on LB agar plates. Flow cytometric analysis was performed on trypsinized m-IC_cl2_ cells following fixation using a FACS Calibur apparatus (BD).

### Statistical analysis

Results for bacterial growth in organ tissues or quantitative RT-PCR show counts for individual animals plus the median. The One-way ANOVA Kruskal-Wallis test (with Dunn's posttest) and the Mann-Whitney test were employed for statistical analysis of bacterial growth in organ tissue at different time points, comparative analysis of quantitative RT PCR results or bacterial counts after infection with WT *versus* SPI1 mutant *Salmonella* respectively. The GraphPad Prism Software 5.00 was used for statistical evaluation. p values are indicated as follows: ***p<0.001; **p<0.01, and *p<0.05.

## Supporting Information

Figure S1 **Organ count after oral infection of neonate mice.** **(A)** Number of viable bacteria in small intestine, colon, spleen, liver and lung tissue at 2 h p.i. after oral infection with high dose (5×10^5^ CFU) *S.* Typhimurium WT infection.
(TIF)

Figure S2 **Comparative analysis of WT and invasion-deficient Δ** ***inv*** **C** ***Salmonella*** **. (A and B)** Organ counts in small intestine **(A)** and colon **(B)** after oral infection of 1-day-old mice with 10^2^ CFU WT (open circles) or Δ*invC* SPI1 mutant (filled circles) *S.* Typhimurium. The results represent the median values from 3–4 independent experiments (n = 9–15 per group). **(C and D)** 1-day-old C57BL/6 mice were orally infected with high dose (10^5^ CFU) WT (open circles, n = 4 each time point) or isogenic Δ*inv*C SPI1 mutant (filled circles, day 1: n = 5; day 2: n = 8) *S.* Typhimurium. (C) Small intestine (SI), colon as well as **(D)** liver and spleen were obtained at 1 and 2 days p.i., homogenized and the number of viable bacteria was determined by serial dilution and plating. The results represent the median values from two independent experiments. **(E)** Organ counts in caecum and colon after oral infection of 6-week-old streptomycin (20 mg) pretreated mice infected with 2×10^8^ CFU WT (open circles) or isogenic Δ*invC* SPI1 mutant (filled circles) *S.* Typhimurium (n = 6). The results represent the median values from one out of two experiments. **(F)** Flow cytometric analysis of enterocytes isolated at day 4 after infection of 6-week-old adult mice with 5×10^8^ CFU WT or SPI1 defective (Δ*invC*) (GFP^+^) *S.* Typhimurium or uninfected control animals. Cells were gated for the epithelial cell marker EpCAM (APC). One representative data set of three independent experiments is shown and the number of GFP^+^ enterocytes is indicated.
(TIF)

Figure S3 **Age-dependent expression of epithelial host defence effectors.** **(A)** Organ counts in spleen and liver of 1-day-old neonate after oral infection with WT or Δ*fimD* mutant *S.* Typhimurium. The results represent the median values from 2 independent experiments (n = 9–10 per group). **(B)** Quantitative RT-PCR analysis for *Spi-B* mRNA in total enterocytes isolated from 1-day-old mice left uninfected or infected with 10^2^ CFU WT or SPI1 mutant (Δ*invC*) *S.* Typhimurium at 4 days p.i. (n = 3–4 per group). **(C)** Immunostaining for Muc2 (red) in small intestinal tissue sections obtained from 5- and 15-day-old as well as from 6 week-old adult mice. Counterstaining with Phalloidin (green) and Dapi (blue). Bar = 25 µm. **(D)** Quantitative RT-PCR for *Muc3*, *Muc5ac* and *Muc2* mRNA in enterocytes isolated from healthy C57BL/6 mice at the indicated age (n = 3 mice per time point).
(TIF)

Figure S4 **Flow cytometric analysis of isolated enterocytes obtained from 5-day-old neonate mice.** **(A)** Cells were gated as depicted to exclude debris and cell aggregates. **(B and C)** Cells were stained with isotype controls **(B)** or antibodies against the epithelial cell marker EpCAM (APC) and the immune cells marker CD45 (PE) **(C)** and analysed by flow cytometry. Representative images obtained in two independent experiments with each 4 animals per group are shown.
(TIF)

Figure S5 **Confirmation of the non-invasive phenotype of the Δ** ***inv*** **C** ***S.*** **Typhimurium strain.** **(A)** Confluent monolayers of polarized murine intestinal epithelial m-IC_cl2_ cells were infected at a multiplicity of infection (MOI) of 10∶1 with WT *S.* Typhimurium, a SPI1 mutant (Δ*inv*C) or the respective complemented strain (Δ*inv*C *pinv*C) for 1 h at 37°C. Cells were subsequently treated with 100 µg/mL gentamicin for 1 h at 37°C, washed three times, lysed in 0.1% Triton and the number of viable bacteria in cell lysate and inoculum was determined by serial dilution and plating. Data are expressed as gentamicin-protected (intracellular) bacteria relative to the inoculum (%). **(B)** Cells were treated similar to (A). Gentamicin treatment (after 1 h reduced to 20 µg/mL) was extended to 3, 6 and 15 h. Cells were lysed and plated to measure viable bacteria. Data indicate the number of viable gentamicin protected (intracellular) bacteria. **(C)** Flow cytometry analysis of uninfected, *S.* Typhimurium WT infected, or SPI1 mutant (Δ*inv*C) *S.* Typhimurium infected intestinal epithelial m-IC_cl2_ cells 4 hours p.i. The indicated numbers (mean ± SD) represent the results of three independent experiments; the images show one representative experiment.
(TIF)

Figure S6 **Confirmation of the** ***S.*** **Typhimurium immunostaining and cultural detection method.** **(A and B)** *S.* Typhimurium (red) was detected in two consecutive sections cut from the same tissue block obtained 4 days p.i. with **(A)** an anti-GFP antibody or **(B)** a *S.* Typhimurium O-antigen specific rat monoclonal anti-O4/O5 antibody. Bar, 25 µm. Counterstain: E-cadherin (green, in A), autofluorescence (green, in B), Dapi (blue). **(C and D)** 1-day-old mice (n = 6–7) were orally infected with 10^2^ CFU *S.* Typhimurium carrying the GFP expression plasmid (pGFP) and the viable organ count in liver and spleen tissue was determined at **(C)** 2 and **(D)** 4 days p.i. by dilution and plating on LB agar plates without supplement (−) or supplemented with 100 µg/mL ampicillin (+).
(TIF)
