# Correction: Age-Dependent Enterocyte Invasion and Microcolony Formation by *Salmonella*

**DOI:** 10.1371/journal.ppat.1004476

**Published:** 2014-09-25

**Authors:** The PLOS Pathogens Staff

The fourth author's name is spelled incorrectly. The correct name is: Frederik Gohde. The citation remains the same.
