# Supplementary figures and images for: Age-Dependent Enterocyte Invasion and Microcolony Formation by Salmonella
Source: PLoS Pathog. 2014 Sep 11;10(9):e1004385. doi: 10.1371/journal.ppat.1004385 (PMC4161480; doi:10.1371/journal.ppat.1004385)

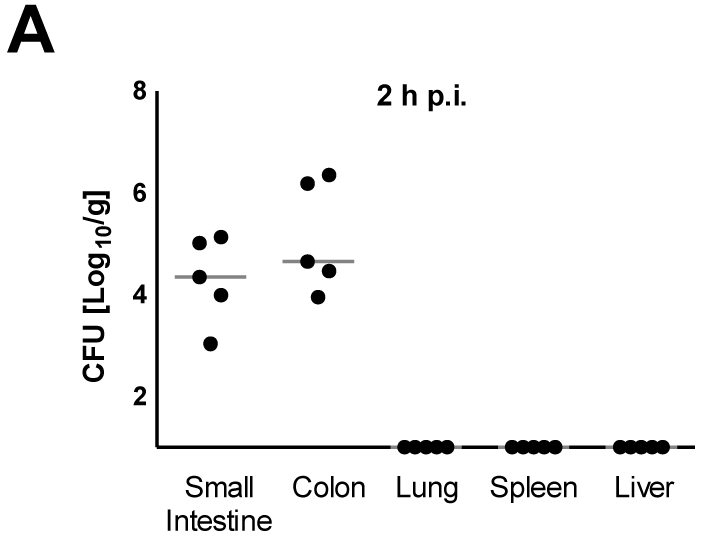

Supplement: Figure S1 — Organ count after oral infection of neonate mice. (A) Number of viable bacteria in small intestine, colon, spleen, liver and lung tissue at 2 h p.i. after oral infection with high dose (5×10^5^ CFU) S. Typhimurium WT infection. (TIF) [file ppat.1004385.s001.tif]

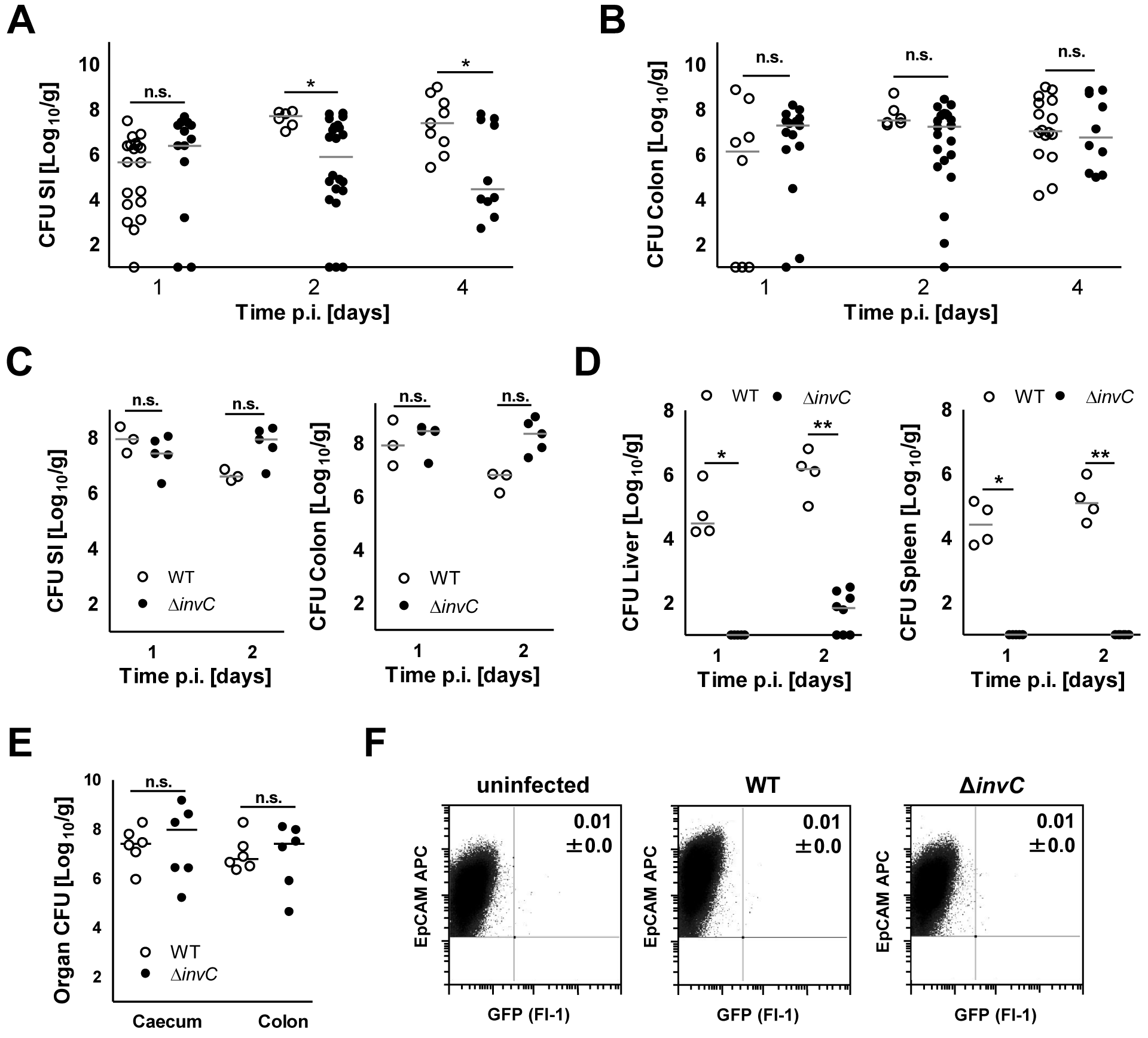

Supplement: Figure S2 — Comparative analysis of WT and invasion-deficient Δ inv C Salmonella . (A and B) Organ counts in small intestine (A) and colon (B) after oral infection of 1-day-old mice with 102 CFU WT (open circles) or ΔinvC SPI1 mutant (filled circles) S. Typhimurium. The results represent the median values from 3–4 independent experiments (n = 9–15 per group). (C and D) 1-day-old C57BL/6 mice were orally infected with high dose (105 CFU) WT (open circles, n = 4 each time point) or isogenic ΔinvC SPI1 mutant (filled circles, day 1: n = 5; day 2: n = 8) S. Typhimurium. (C) Small intestine (SI), colon as well as (D) liver and spleen were obtained at 1 and 2 days p.i., homogenized and the number of viable bacteria was determined by serial dilution and plating. The results represent the median values from two independent experiments. (E) Organ counts in caecum and colon after oral infection of 6-week-old streptomycin (20 mg) pretreated mice infected with 2×10^8^ CFU WT (open circles) or isogenic ΔinvC SPI1 mutant (filled circles) S. Typhimurium (n = 6). The results represent the median values from one out of two experiments. (F) Flow cytometric analysis of enterocytes isolated at day 4 after infection of 6-week-old adult mice with 5×10^8^ CFU WT or SPI1 defective (ΔinvC) (GFP+) S. Typhimurium or uninfected control animals. Cells were gated for the epithelial cell marker EpCAM (APC). One representative data set of three independent experiments is shown and the number of GFP+ enterocytes is indicated. (TIF) [file ppat.1004385.s002.tif]

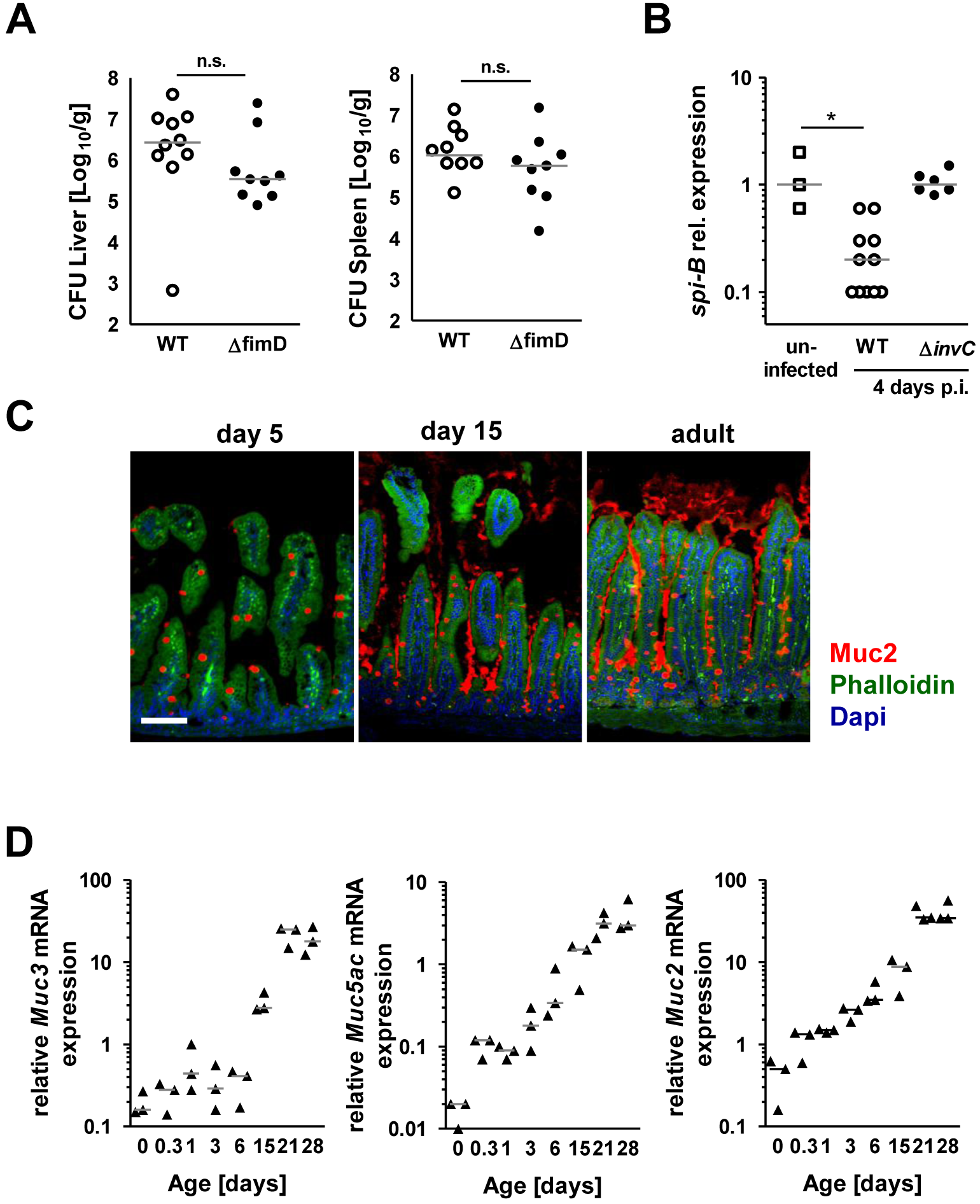

Supplement: Figure S3 — Age-dependent expression of epithelial host defence effectors. (A) Organ counts in spleen and liver of 1-day-old neonate after oral infection with WT or ΔfimD mutant S. Typhimurium. The results represent the median values from 2 independent experiments (n = 9–10 per group). (B) Quantitative RT-PCR analysis for Spi-B mRNA in total enterocytes isolated from 1-day-old mice left uninfected or infected with 102 CFU WT or SPI1 mutant (ΔinvC) S. Typhimurium at 4 days p.i. (n = 3–4 per group). (C) Immunostaining for Muc2 (red) in small intestinal tissue sections obtained from 5- and 15-day-old as well as from 6 week-old adult mice. Counterstaining with Phalloidin (green) and Dapi (blue). Bar = 25 µm. (D) Quantitative RT-PCR for Muc3, Muc5ac and Muc2 mRNA in enterocytes isolated from healthy C57BL/6 mice at the indicated age (n = 3 mice per time point). (TIF) [file ppat.1004385.s003.tif]

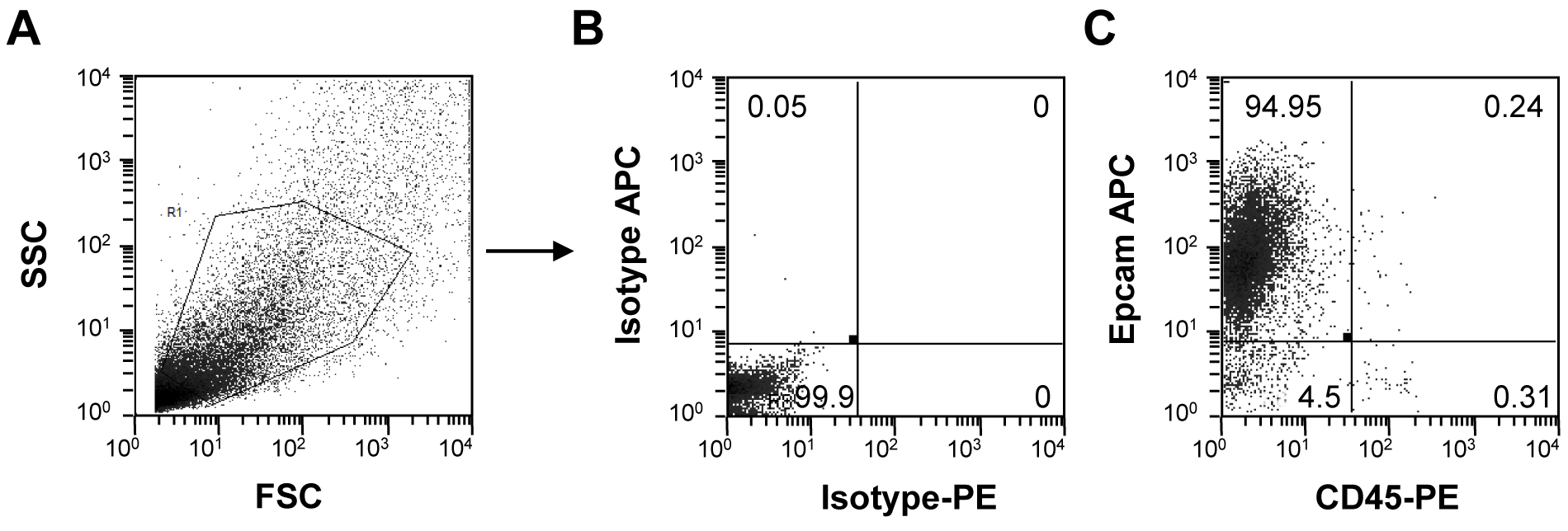

Supplement: Figure S4 — Flow cytometric analysis of isolated enterocytes obtained from 5-day-old neonate mice. (A) Cells were gated as depicted to exclude debris and cell aggregates. (B and C) Cells were stained with isotype controls (B) or antibodies against the epithelial cell marker EpCAM (APC) and the immune cells marker CD45 (PE) (C) and analysed by flow cytometry. Representative images obtained in two independent experiments with each 4 animals per group are shown. (TIF) [file ppat.1004385.s004.tif]

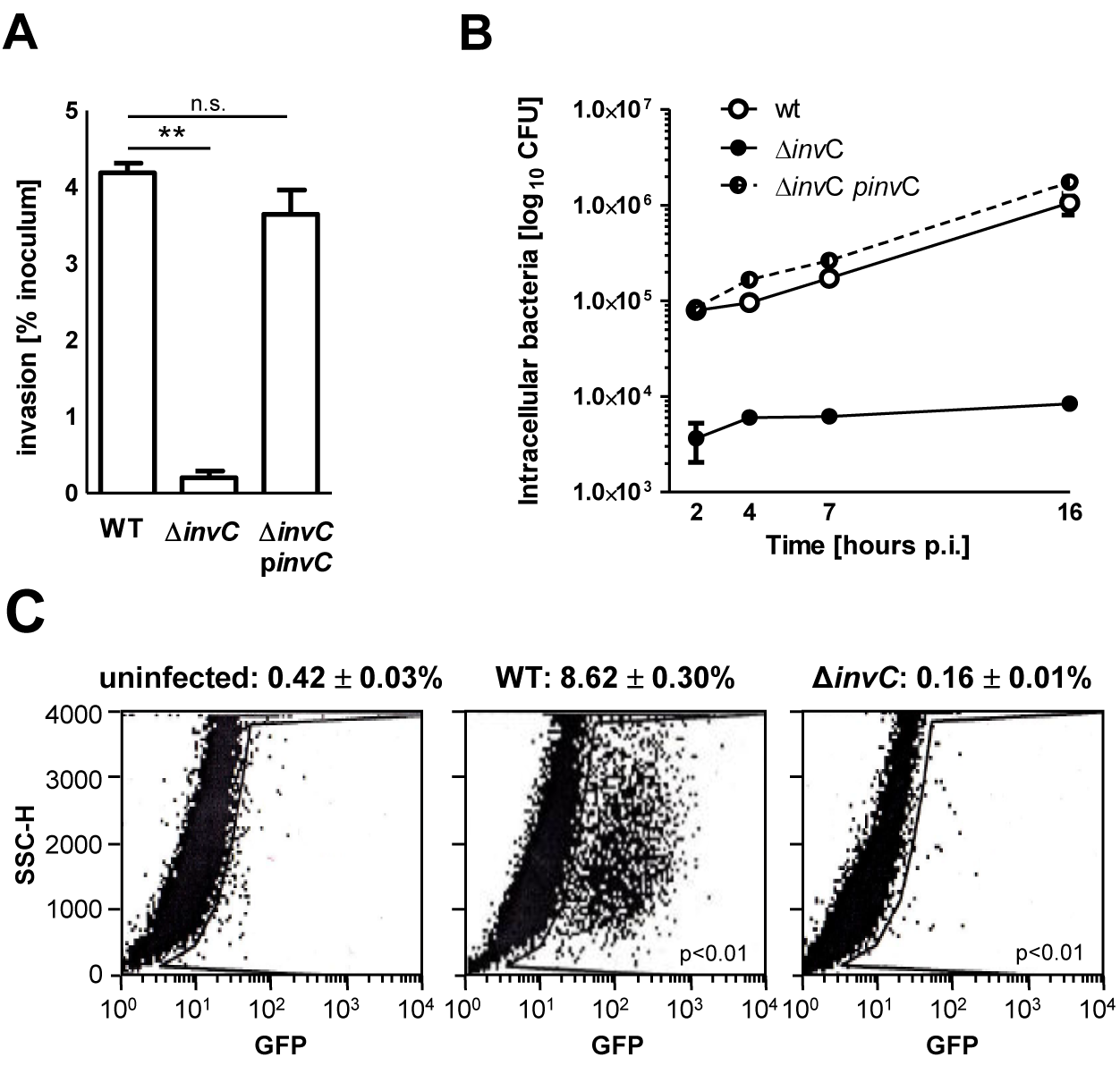

Supplement: Figure S5 — Confirmation of the non-invasive phenotype of the Δ inv C S. Typhimurium strain. (A) Confluent monolayers of polarized murine intestinal epithelial m-ICcl2 cells were infected at a multiplicity of infection (MOI) of 10∶1 with WT S. Typhimurium, a SPI1 mutant (ΔinvC) or the respective complemented strain (ΔinvC pinvC) for 1 h at 37°C. Cells were subsequently treated with 100 µg/mL gentamicin for 1 h at 37°C, washed three times, lysed in 0.1% Triton and the number of viable bacteria in cell lysate and inoculum was determined by serial dilution and plating. Data are expressed as gentamicin-protected (intracellular) bacteria relative to the inoculum (%). (B) Cells were treated similar to (A). Gentamicin treatment (after 1 h reduced to 20 µg/mL) was extended to 3, 6 and 15 h. Cells were lysed and plated to measure viable bacteria. Data indicate the number of viable gentamicin protected (intracellular) bacteria. (C) Flow cytometry analysis of uninfected, S. Typhimurium WT infected, or SPI1 mutant (ΔinvC) S. Typhimurium infected intestinal epithelial m-ICcl2 cells 4 hours p.i. The indicated numbers (mean ± SD) represent the results of three independent experiments; the images show one representative experiment. (TIF) [file ppat.1004385.s005.tif]

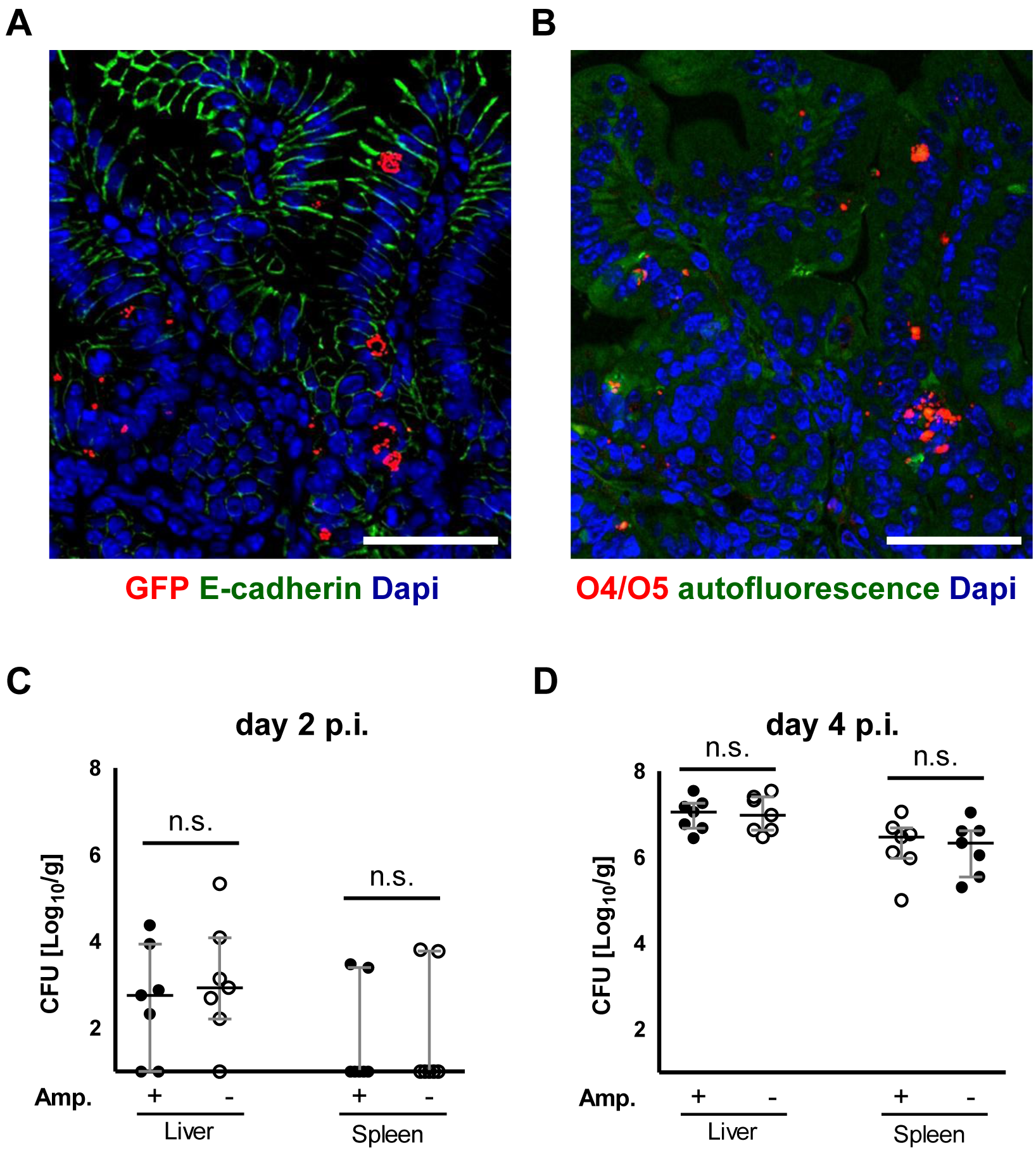

Supplement: Figure S6 — Confirmation of the S. Typhimurium immunostaining and cultural detection method. (A and B) S. Typhimurium (red) was detected in two consecutive sections cut from the same tissue block obtained 4 days p.i. with (A) an anti-GFP antibody or (B) a S. Typhimurium O-antigen specific rat monoclonal anti-O4/O5 antibody. Bar, 25 µm. Counterstain: E-cadherin (green, in A), autofluorescence (green, in B), Dapi (blue). (C and D) 1-day-old mice (n = 6–7) were orally infected with 102 CFU S. Typhimurium carrying the GFP expression plasmid (pGFP) and the viable organ count in liver and spleen tissue was determined at (C) 2 and (D) 4 days p.i. by dilution and plating on LB agar plates without supplement (−) or supplemented with 100 µg/mL ampicillin (+). (TIF) [file ppat.1004385.s006.tif]
